# Ventilator auto‐triggering by cardiac electrical activity during noninvasive ventilation with neurally adjusted ventilatory assist

**DOI:** 10.1002/ccr3.1590

**Published:** 2018-05-15

**Authors:** Yu Inata, Muneyuki Takeuchi

**Affiliations:** ^1^ Department of Intensive Care Medicine Osaka Women’s and Children’s Hospital Izumi Japan

**Keywords:** auto‐triggering, neurally adjusted ventilatory assist, noninvasive ventilation, patient‐ventilatory synchrony

## Abstract

Neurally adjusted ventilatory assist (NAVA), by capturing the electrical activity of the diaphragm, improves patient‐ventilator synchrony. It is, however, not completely immune from auto‐triggering by cardiac electrical activity as illustrated in this case. Stringent observation of respiratory rate and vigilance for this phenomenon is warranted when using NAVA.

## 1 QUESTION

**Figure 1:**
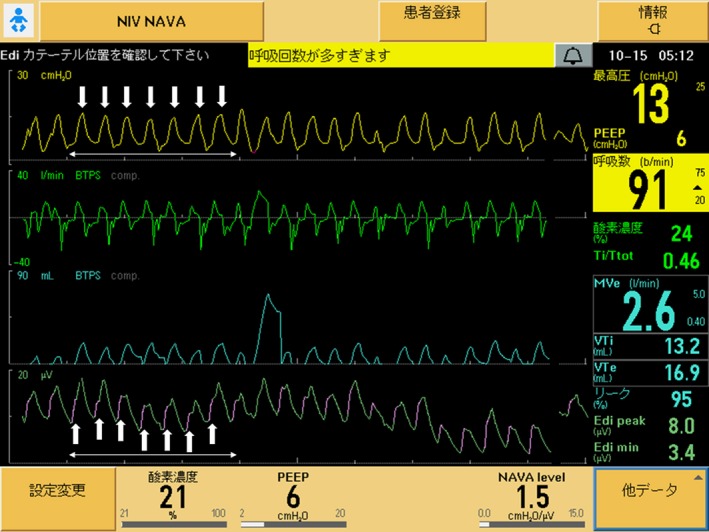
Ventilator graphics at the time of auto‐triggering by cardiac electrical activity. The waveforms at the bottom of the screen (displayed in blue‐green and purple), which should be derived from electrical activity of the diaphragm (Edi), were reflecting cardiac electrical activity. In a period of 3‐s (double‐headed arrows), there are 7 waves of electrical activity (up arrows) and 7 waves of delivered breaths to the patient (down arrows); the rate is approximately 140/min that corresponds to the heart rate of the patient. Purple color in the wave upstroke of waveforms at the bottom indicates that delivered breaths were being triggered by electrical activity: cardiac electrical activity instead of Edi in this case

**Figure 2:**
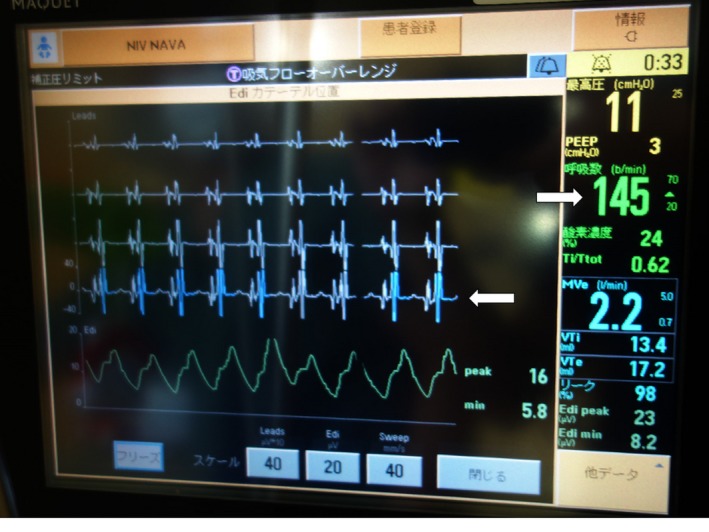
The Edi catheter positioning screen. There are 4 leads that display electrocardiogram signals. The second and third leads should be highlighted in blue for ideal positioning of the catheter. In this case, the catheter position was shallower than ideal, as indicated by blue color only at the bottom of 4 leads (a left arrow). The green waveforms at the bottom of the screen, which should be derived from electrical activity of the diaphragm, correspond to cardiac electrical activity. Note that the ventilator respiratory rate on the screen is 145 b/min (displayed in green color, indicated by a right arrow) because of auto‐triggering by cardiac electrical activity

An 8‐month‐old female with tetralogy of Fallot, pulmonary valve atresia, and major aortopulmonary collateral arteries was admitted to the intensive care unit after unifocalization and palliative right ventricular outflow tract reconstruction. She was extubated and supported on NIV‐NAVA. After a few weeks of NIV‐NAVA support, end‐expiratory positive pressure, NAVA level, and electrical activity of the diaphragm (Edi) trigger were set at 5 cmH_2_O, 1.5 cmH_2_O/μV, and 0.5 μV, respectively, when suddenly the ventilator alarm signaled a high respiratory rate. The respiratory rate shown on the ventilator screen was approximately 140 breaths per minute (b/min) despite observed breathing at a rate of 60 b/min. The waveforms of Edi appeared rhythmic, with each wave triggering the ventilator (Figure 1). The Edi catheter positioning screen was reviewed (Figure 2). What happened?

## 2 ANSWER

By examining the patient and the Edi catheter positioning screen, the Edi catheter position was found to be inadvertently withdrawn and the Edi waves were corresponding to cardiac electrical activity (Figure 2). By advancing the Edi catheter deeper, capturing of Edi was restored, so was patient‐ventilator synchrony. In the NAVA mode, signals from the Edi catheter are filtered to remove electrical contamination by various sources including the heart.^1^ While the detection of cardiac electrical activity by Edi catheter in a patient with atrial fibrillation has been reported,^2^ ventilator “auto‐triggering” by cardiac electrical activity has not been described.

## AUTHOR CONTRIBUTIONS

YI: collected and analyzed data, drafted, and revised the manuscript. MT: helped YI collect and analyze data, provided intellectual input, and revised the manuscript; both authors read and approved the final manuscript.

## CONFLICTS OF INTEREST

The authors declare that they have no conflict of interest. No funding or financial support was received.
